# Non-Coding RNAs: New Dawn for Diabetes Mellitus Induced Erectile Dysfunction

**DOI:** 10.3389/fmolb.2022.888624

**Published:** 2022-06-22

**Authors:** Wenchao Xu, Hongyang Jiang, Jihong Liu, Hao Li

**Affiliations:** ^1^ Department of Urology, Tongji Hospital, Tongji Medical College, Huazhong University of Science and Technology, Wuhan, China; ^2^ Institute of Urology, Tongji Hospital, Tongji Medical College, Huazhong University of Science and Technology, Wuhan, China

**Keywords:** erectile dysfunction, diabetes mellitus, non-coding RNA, miRNA, lncRNA

## Abstract

Erectile dysfunction (ED) is a common sexual dysfunction in males, with multifactorial alterations which consist of psychological and organic. Diabetes mellitus (DM) induced erectile dysfunction (DMED) is a disconcerting and critical complication of DM, and remarkably different from non-diabetic ED. The response rate of phosphodiesterase type 5 inhibitor (PDE5i), a milestone for ED therapy, is far from satisfactory in DMED. Unfortunately, the contributing mechanisms of DMED remains vague. Hence, It is urgent to seek for novel prospective biomarkers or targets of DMED. Numerous studies have proved that non-coding RNAs (ncRNAs) play essential roles in the pathogenesis process of DM, which comprise of long non-coding RNAs (lncRNAs) and small non-coding RNAs (sncRNAs) like microRNAs (miRNAs), PIWI-interacting RNAs (piRNAs) and circular RNAs (circRNAs). However, the implications of ncRNAs in DMED are still understudied. This review highlights the pathophysiology of DMED, summarizes identified mechanisms of ncRNAs associated with DMED and covers the topic of perspectives for ncRNAs in DMED.

## Introduction

Diabetes mellitus (DM) has evolved as one of the most severe and widespread chronic conditions, leading in life threatening, debilitating and high-cost complications, as well as a reduction in life expectancy (Heald et al., 2020). Over the last three decades, the worldwide prevalence of DM has risen fast and reached pandemic proportions, with the 10th edition of the International Diabetes Federation indicating a prevalence of 536.6 million individuals (Sun et al., 2022). However, lowering diabetes mortality as a consequence of improved medical treatment, as well as rises in diabetes incidence in certain countries as a result of rising prevalence of diabetes risk factors, particularly obesity, are also significant drivers of increased prevalence (Magliano et al., 2019; Chan et al., 2020). It was concurrent with an increase in the prevalence of microvascular and macrovascular complications of DM (Graves and Donaghue, 2020).

Erectile dysfunction (ED) is a common and frequently occurring disease, characterized as the inability to attain or sustain an erection adequate for enjoyable sexual performance (Shamloul and Ghanem, 2013; Yafi et al., 2016). The incidence of ED is reported to be as high as 75% in diabetics, more than three times that in non-diabetics (Thorve et al., 2011). Numerous pathological alterations affecting the corpus cavernosum, including as endothelial dysfunction and nitric oxide (NO) bioactivity, have been implicated in the development of ED (Lue, 2000). Sildenafil citrate, the first successful oral medication, is a selective phosphodiesterase type 5 inhibitor (PDE5i). It is generally acknowledged as the first-line treatment for erectile dysfunction (ED). The PDE5i were developed based on the crucial function of NO in penile cavernous smooth muscle relaxation, which results in effective erections in 63% of men with general ED. However, its response rate in DMED is far from optimal, only about 44% in patients with inadequate glycemic control (Liu et al., 2010). As a result, DMED is a current research hotspot. Regrettably, the processes behind DMED remains vague.

Profiling of various cell lines using high-throughput sequencing found that 74% of the human genome is transcribed, although only 2% of it contains protein-coding genes (Esteller, 2011; Djebali et al., 2012). As a result, the vast majority of the human transcriptome is composed of non-coding RNAs (ncRNAs). ncRNAs are characterized as regulatory RNAs that do not comprise a protein-coding region (Carninci et al., 2005). Based on their length, ncRNAs are classified as small non-coding RNAs (200 nt) and long non-coding RNAs (lncRNAs, >200 nt) (Matsui and Corey, 2017). However, according to localization and functions, ncRNAs can be divided into lncRNAs, miRNAs, circular RNAs (circRNAs), small nucleolar RNAs (snoRNAs), small nuclear RNAs (snRNAs) and PIWI-interacting RNAs (piRNAs). So, it is vital not to be dogmatic with regard to terminology, as this may impede data interpretation and mechanistic understanding (Matsui and Corey, 2017). In recent years, it has become clearer that ncRNAs play a critical role in normal development and physiology, as well as in diseases (Chi et al., 2021; de Goede et al., 2021). The functional significance of ncRNAs is most clear in the case of miRNAs. It has been demonstrated that human illnesses frequently exhibit epigenetic and genetic alterations in miRNAs and their processing machinery (Ji and Guo, 2019; Mori et al., 2019; Agbu and Carthew, 2021). However, miRNAs are just the tip of the iceberg; other ncRNAs also have a role in the development of a variety of other illnesses (Bridges et al., 2021). There is mounting evidence that ncRNAs grease the wheels of the development of DM and associated complications (Feng et al., 2019; Thomas et al., 2019). There is considerable interest in medicinal methods that target these ncRNAs disruptions (Matsui and Corey, 2017).

Here, we begin by discussing the pathophysiology of DMED. On top of that, we consider in greater detail the growing evidence for the roles of miRNAs and lncRNAs, which have been implicated in several cellular processes. Finally, this Review covers the potential for adopting innovative treatment techniques to target these ncRNAs disruptions.

## Diabetes Mellitus Induced Erectile Dysfunction

**FIGURE 1:**
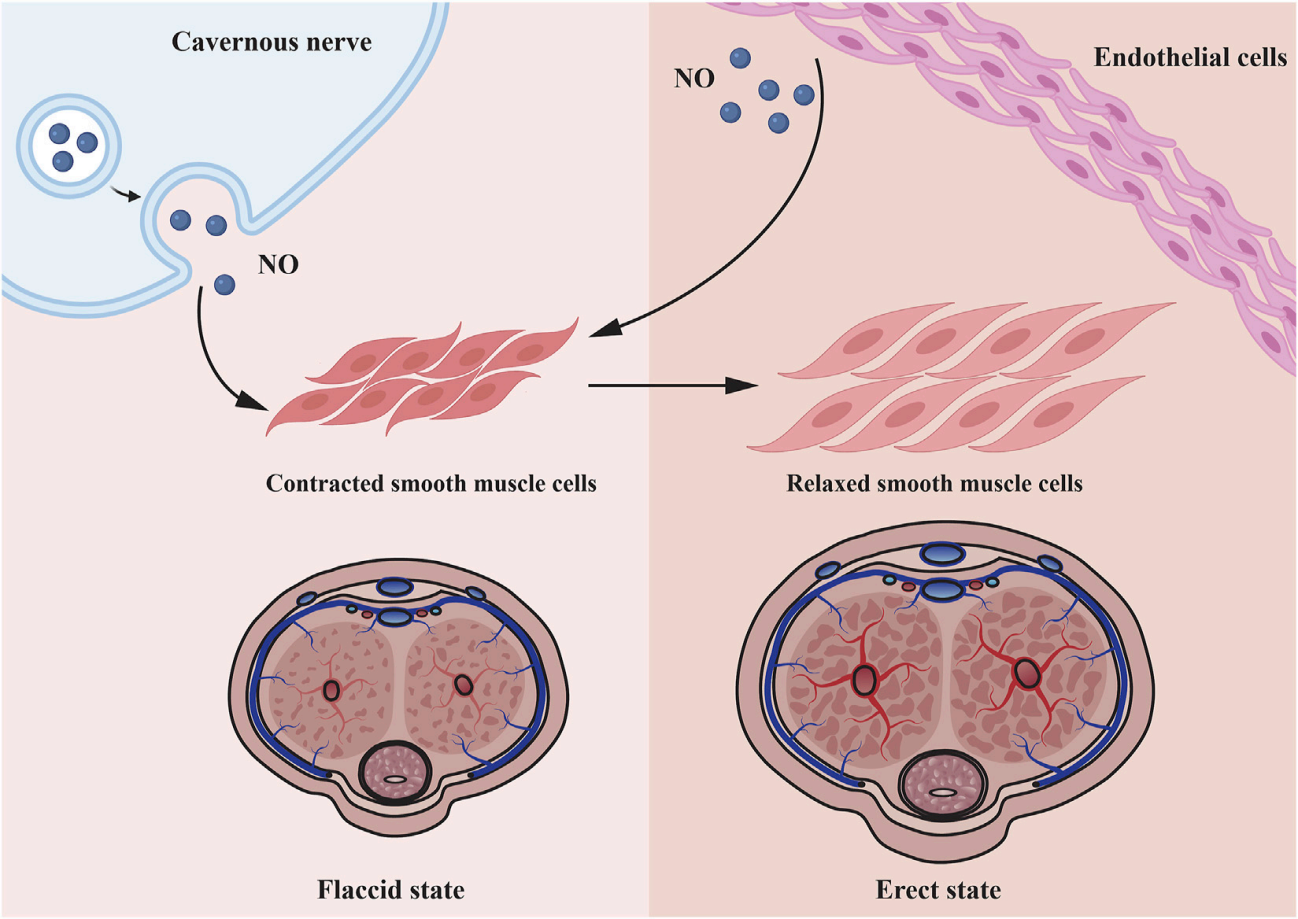
Microscopic mechanisms underlying penile smooth muscle relaxation. Cavernous nerve and endothelial cell all can secrete NO to relax corpus cavernosum smooth muscle cells. As the smooth muscle relaxes, blood fills the lacunar spaces, resulting to compression of the subtunical venules, thereby restricting the venous outflow.

DMED is considered to have a multifactorial etiology (Malavige and Levy, 2009). Numerous physical causes are assumed to be essential, but psychological and relationship concerns frequently coincide (Muneer et al., 2014; Najari and Kashanian, 2016). During an erection, increased blood flow to penile corpora cavernosa is caused by nerve signals that relax the vascular and corpus cavernosum smooth muscle cells (CCSMCs) (McMahon, 2019). This is primarily mediated by NO, which is generated by parasympathetic nonadrenergic noncholinergic neurons and cholinergic neurons when they stimulate vascular endothelial cells (VECs), initiating a biochemical cascade that leads in smooth muscle relaxation and vasodilation (Udelson, 2007; Mitidieri et al., 2020). Increasing blood flow compresses subtunical venules to impede venous return, hence preserving the erection (Yafi et al., 2016) (Figure 1). The pathophysiology of DMED is primarily characterized by functional and structural alterations in two dimensions. Early alterations that were mostly functional in nature: long-term hyperglycemia stimulation results in dysfunction of the penile artery and endothelium (Castela and Costa, 2016), increased reactive oxygen species (Yuan et al., 2020) and decreased NO production and bioavailability (Zhou et al., 2021). Simultaneously, the generation of contractile substances like as angiotensin and endothelin raise the concentration of Ca^2+^ in CCSMCs and activates RohA/ROCK signaling pathways (Yuan et al., 2020), resulting in the impairment of smooth muscle’s diastolic function. As the disease progresses, a high concentration of reactive oxygen species and fibrogenic factors are produced in the cavernosum of the penis, resulting in excessive endothelial cell apoptosis, smooth muscle atrophy, structural changes such as fibrosis, and eventually cavernous venous closure dysfunction (Li et al., 2021).

## miRNAs in Diabetes Mellitus Induced Erectile Dysfunction

For a long period of time, the function of ncRNAs were unknown, and they were not regarded as critical as transcripts of protein-coding genes. Our current understanding of the functions which some research hotspots in ncRNAs, like miRNAs and lncRNAs, play within cells is expanding rapidly, and we now know more than ever before about their impact on a wide range of physiological processes and illnesses (Pandolfini et al., 2019). One of the most well-studied classes of ncRNAs is miRNAs, which function to silence genes post-transcriptionally by interfering with mRNA translation into proteins. Although the discovery is only a few years old, it is of great significance for our understanding of post-transcriptional regulation of genes (Bartel, 2018). In contrast to certain miRNAs, which target particular genes, others might act as master regulators of processes, regulating hundreds of genes concurrently and cooperating with other miRNAs (Agbu and Carthew, 2021).

**FIGURE 2:**
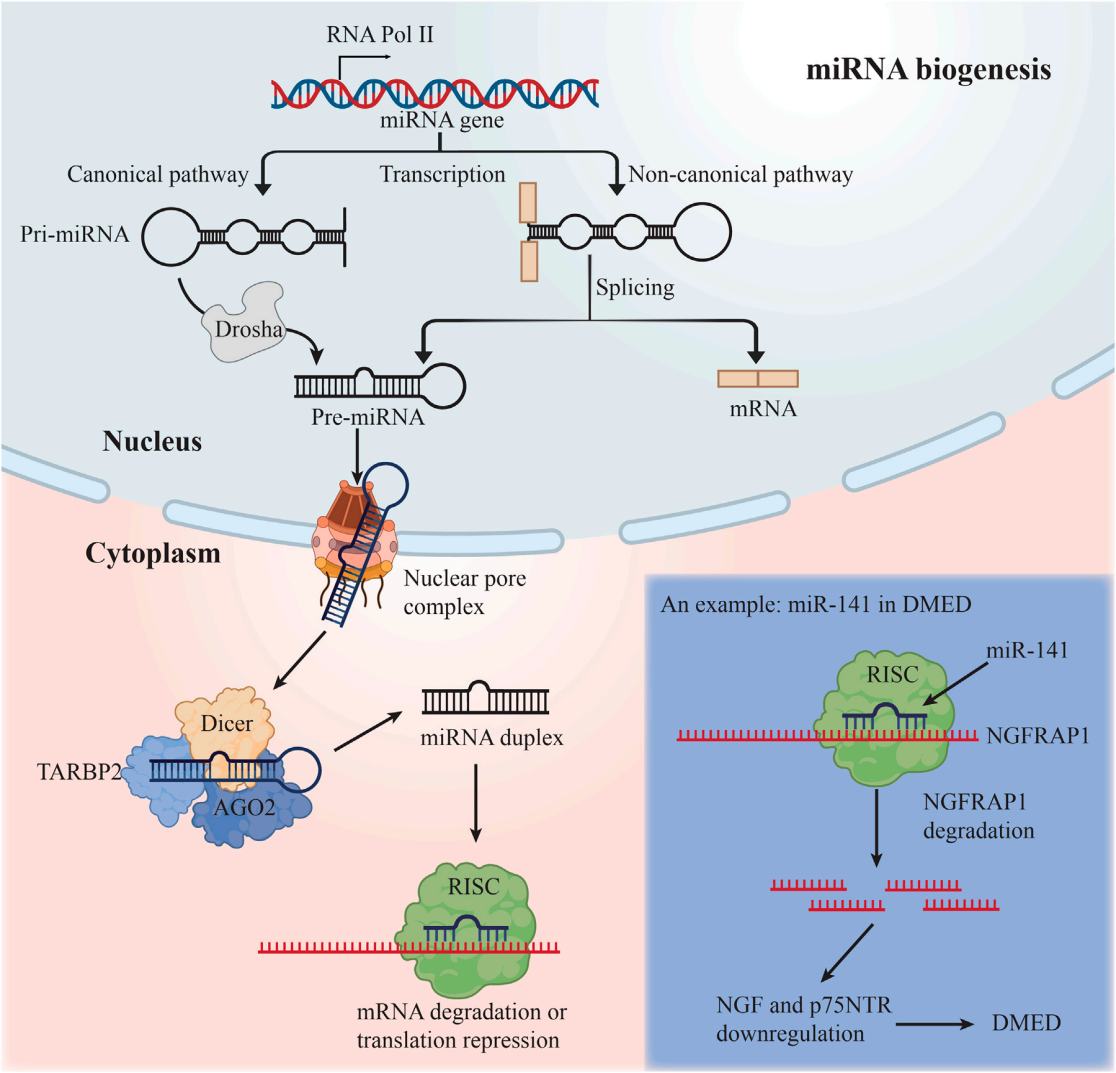
The biogenesis of miRNAs and an example of miRNA in DMED.

In order to produce mature miRNAs, the RNase III enzymes Drosha and Dicer participate in a multi-step biogenesis process (Krol et al., 2010) (Figure 2). The Dicer–TARBP2 (TAR RNA-binding protein 2) complex loads these molecules into a member of the Argonaute protein subfamily, which serves as the catalytic endonuclease component, to create the RNA-induced silencing complex (RISC). RISC recognizes a complementary region in the 3′UTR of the targeted mRNA and guides the regulation of mRNA. There is a great deal of control over the loading of miRNAs into RISC (Gebert and MacRae, 2019) and the operation of the miRNA machinery itself (Krol et al., 2010). miRNAs impede the translation of mRNA by mRNA degradation and translation start inhibition.

The first miRNA profile for DMED patients was conducted by (Jiang et al., 2015). They assessed the serum miRNA content in normal individuals, non-diabetic ED, and DMED patients, and discovered that the serum miRNA content of DMED patients was considerably elevated for miR-93, miR-320, and miR-16 (Jiang et al., 2015). Recently, Xu et al. (2021) applied miRNA sequencing form the serum of diabetic and DMED patients and discovered that the serum levels of let-7e-5p, miR-30d-5p, miR-199b-5p, and miR-342-3p were considerably higher in DMED patients. It is indicated the presence of miRNAs in serum may aid in the early detection of DMED.

Wei et al. (2011) discovered decreased expression of miR-145 in the corpus cavernosum of DMED rats, establishing the first relationship between miRNAs and DMED. It wasn’t until 2016 that the first miRNA profile for erectile dysfunction in mice with type 2 diabetes was published. eNOS/cGMP/PKG pathway and the contraction of vascular smooth muscle may all be affected by specific miRNAs, such as miR-18a, miR206, and miR-122 and miR-133. The researchers hypothesized that these miRNAs could have a significant impact on the endothelium and smooth muscle in the corpus cavernosum (Pan et al., 2016). Additionally, miR-328 antagomir has been shown to enhance erectile performance in diabetic rats by downregulating the expression of advanced glycation end products and increasing the levels of DKK3, cGMP and eNOS (Li et al., 2017). According to a recent research, elevation of NGFRAP1, NGF, and p75NTR in DMED is related with decreased expression of miR-141, and the overexpression of miR-141 can improve erectile function in DMED rats (Wen et al., 2018). Furthermore, Wen et al. (2019) discovered that DMED rats had significantly higher levels of miR-205 expression than normal rats, and further research revealed that miR-205 could directly act on androgen receptors, causing fibrosis and apoptosis of corpus cavernosum smooth muscle cells (CCSMCs), ultimately leading to DMED. This work presented a novel method by which miRNAs influence AR expression in target cells, which is predicted to give a new technique for treating DMED in the clinic by modulating AR expression. As demonstrated by Huo et al. (2020a), the pathophysiology of DMED rats may be connected to the downregulation of the miR-874-3p after an increase in methylation at the promoter region, followed by the upregulation of the Nupr1/Chop pathway, thereby speeding the death of CCSMCs in rats and impairing erectile function. Bioinformatics is a critical tool for predicting the presence of ED-related miRNAs. Kazemi et al. (2021) attempted to identify the conserved site for miRNAs, and revealed that the conserved miR-29-3p binding site is found in the 3’UTR of genes related with ED.

Research by Gonçalves et al. (2018) and Tiraboschi et al. (2021) created three distinct types of ED models in rats: diabetic, alcoholic, or both. Their outcomes, however, are distinct. Gonçalves et al. (2018) discovered that in alcoholic and alcoholic–diabetic conditions, reduced expression of miR-199 and miR-155 boosted endothelin receptor (ETA and ERB) expression. Then, in another study, miR-15b, miR-16, miR-138, miR-221, and miR-222 could be considered prospective biomarkers for diabetic alcoholic ED (Tiraboschi et al., 2021).

Adipose tissue-derived stem cells (ADSCs) can enhance erectile function in diabetic rats by altering the microarchitecture of the corpus cavernosum. A second option is ADSC-exosomes, which is derived from ADSCs. Zhu et al. (2018) showed that ADSCs-Exosomes had a greater expression of various pro-angiogenic or antifibrotic miRNAs, including miR-126, miR-130a, miR-132, let7b, and let7c, than ADSCs. Ouyang et al. (2019) revealed that some pro-angiogenic miRNAs, including miR-21-5p, the let-7 family, the miR-10 family, the miR-30 family, and miR-148a-3p, are expressed at a greater level in ADSCs-exosomes than in ADSCs. Taken together, these distinctions between ADSCs and ADSCs-exosomes may contribute to ADSC-exosomes superior therapeutic efficacy over ADSCs. Furthermore, Huo et al. (2020b) verified that exosomal miR-21-5p generated from bone marrow-derived stem cells (BMSCs) inhibited PDCD4 expression and ED in rats with DM.

Table 1 summarizes the miRNAs in DMED and their possible mechanism. Clearly, the majority of miRNAs appear to be intimately linked to smooth muscle contraction or apoptosis. Thus, it is beneficial to regulate miRNAs in order to optimize smooth muscle performance.

**TABLE 1:** miRNAs in DMED.

Study (Author, year)	miRNA name	Specimen	Function
Jiang et al. (2015)	miR-93, miR-320, and miR-16	blood (human)	prospective markers
Xu et al. (2021)	let-7e-5p, miR-30d-5p, miR-199b-5p and miR-342-3p	blood (human)	prospective markers
Wei et al. (2011)	miR-145	Penis (rat)	Prospective markers
Pan et al. (2016)	miRNA-18a, miRNA-206, miRNA-122 and miRNA-133	penis (mouse)	regulate eNOS/cGMP/PKG pathway and the contraction of vascular smooth muscle
Li et al. (2017)	miRNA-328	penis (rat)	increases AGEs and inhibits DKK3, cGMP and eNOS.
Gonçalves et al. (2018)	miRNA-199 and miRNA-155	blood and penis (rat)	inhibits ETA and ETB receptors
Wen et al. (2018)	miRNA-141	penis (rat)	inhibits the NGF/p75NTR signaling via NGFRAP1
Zhu et al. (2018)	miR-126, miR-130a, miR-132, let-7b and let-7c	penis (rat)	enhances the treatment of ADSC-Exosomes in DMED.
Huo et al. (2019)	miR-328a-5p	penis (rat)	competitive endogenous RNA for lncRNA MIAT.
Ouyang et al. (2019)	miR-21-5p, the let-7 family, the miR-10 family, the miR-30 family, and miR-148a-3p	penis (rat)	enhances the treatment of ADSC-Exosomes in DMED.
Wen et al. (2019)	miR-205	penis (rat)	acts on androgen receptors, causing fibrosis and apoptosis of CCSMCs
Huo et al. (2020a)	miR-874-3p	penis (rat)	inhibits the Nupr1/Chop pathway
Huo et al. (2020b)	miR-21-5p	penis (rat)	inhibited PDCD4 expression, enhances the treatment of ADSC-Exosomes in DMED.
Kazemi et al. (2021)	miR-29-3p	penis (rat)	prospective marker
Tiraboschi et al. (2021)	miR-15b, miR-16, miR-138, miR-221 and miR-222	blood and penis (rat)	prospective marker

eNOS, endothelial nitric oxide synthase; cGMP, cyclic guanosine monophosphate; PKG, protein kinase G; AGEs, advanced glycation end products; DKK3, dickkopf-3; ET, endothelin receptor; NGF, nerve growth factor; p75NTR, p75 neurotrophin receptor; NGFRAP1, nerve growth factor receptor–associated protein 1; ADSCs, Adipose tissue-derived stem cells; MIAT, myocardial infarction-associated transcript; CCSMCs, corpus cavernosum smooth muscle cells.

## lncRNAs in Diabetes Mellitus Induced Erectile Dysfunction

IncRNAs has some structural characteristics of mRNA, including 3′poly(A) tails and terminal 5′caps, but lacks open reading frame, so it does not encode protein (Statello et al., 2020). Rapid advancements in high-throughput sequencing technology have resulted in the identification of an increasing number of differentially expressed lncRNAs (Fang et al., 2018; Uszczynska-Ratajczak et al., 2018). They are abundant in tissues, urine, and serum, with expression patterns that vary according to cell type, tissue, and developmental stage (Encode Project Consortium, 2012). lncRNAs are functionally involved in a variety of complicated biological processes via a variety of methods. These include transcription factor titration (Luo et al., 2016), splicing modification, miRNA sponging (Fatica and Bozzoni, 2014), and chromatin modification enzyme recruitment (Isoda et al., 2017; Mumbach et al., 2019). Additionally, there is mounting evidence that lncRNAs have a role in the onset and progression of a variety of disorders, including cardiovascular diseases (Wang and Sun, 2020), metabolic syndrome (Sun L.-Y. et al., 2018), renal fibrosis (Yang et al., 2019), and malignancies (Tan et al., 2021).

Huo et al. (2019) has demonstrated that the lncRNA myocardial infarction-associated transcript (MIAT) is a competitive endogenous RNA for miR-328a-5p. Additionally, by suppressing miR-328a-5p, excessive levels of MIAT/lipoprotein lipase (LPL) pathway can induce damage and death in vascular endothelial cells (VECs). Thus, the MIAT/miR-328a-5p/LPL signaling pathway may provide therapeutic targets for DMED attenuation if the expression of the three critical sites is reversed.

Although stem cell therapy is widely accepted as an effective treatment for erectile dysfunction, the underlying processes are still a mystery. By promoting the degradation of FOXM1 protein and decreasing VEGF expression, lncRNA MEG3 plays a vital role in the differentiation of bone marrow-derived mesenchymal stem cells (BM-MSCs) into VECs (Sun X. et al., 2018). VEGF expression was upregulated by another lncRNA, MALAT1, which worked as a sponge for miR-206 and aided in the differentiation process (Sun et al., 2020).

In general, researchers specializing in the pathophysiology of DMED do not appear to devote sufficient attention to lncRNAs (Chen et al., 2020). Other studies, on the other hand, may discover the mechanism through which BM-MSCs increase erectile function, hence improving therapy options for DMED.

## Therapies Targeting ncRNAs

**FIGURE 3:**
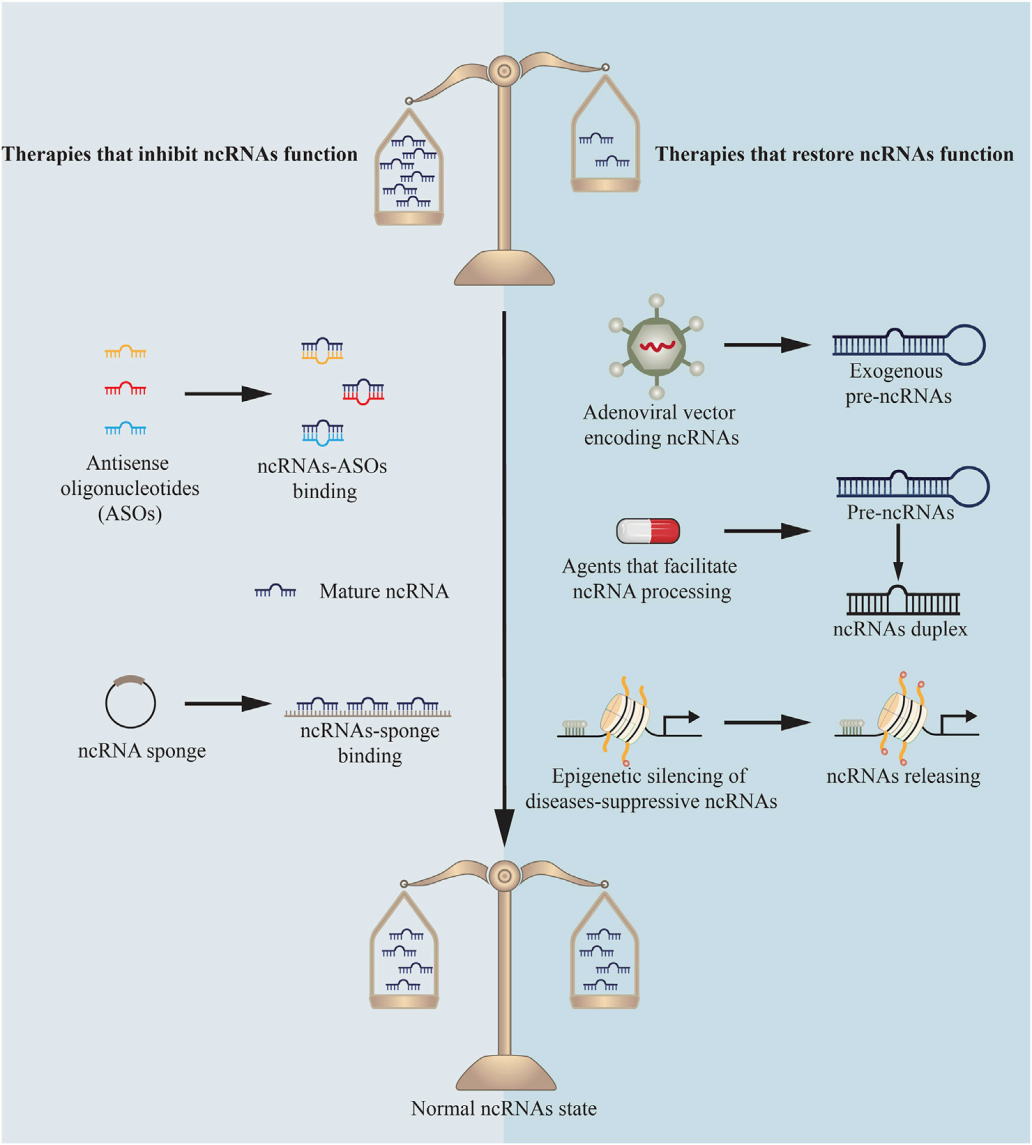
Therapies targeting ncRNAs.

NcRNAs and the protein machinery involved in their synthesis or function have been identified as targets for innovative treatment strategies and tested in clinical practices (Figure 3). Until now, the majority of research in this field has focused on miRNAs’ function in cancer to repress tumor growth (Matsui and Corey, 2017; Mollaei et al., 2019). Additionally, antisense oligonucleotides (ASOs) complementary to miR-122 are being developed to treat Hepatitis C virus (Thakral and Ghoshal, 2015) and miR-21 mimics are being applied to treat cutaneous and pulmonary fibrosis (Matsui and Corey, 2017). While this research is still in its infancy, there is tremendous interest in extending comparable methodologies to different types of diseases, both for miRNAs and other ncRNAs.

### Therapies That Inhibit miRNAs Function

Because miRNAs control their targets via base pairing, ASOs have been developed to therapeutically decrease miRNA activity. Through base pair complementarity, ASOs block miRNA targets. Three distinct classes of ASOs have been developed: locked nucleic acids (LNAs) (Elmén et al., 2008), anti-miRNA oligonucleotides (AMOs) (Yu et al., 2020), and antagomirs (Krützfeldt et al., 2005). Each of these classes incorporates a variety of chemical changes to boost stability and efficacy. For example, antagomirs were first synthesized as miRNA silencing agents in 2005, and they are chemically modified, cholesterol-conjugated oligonucleotides complementary to or the same as miRNAs (Krützfeldt et al., 2005; Mohr and Mott, 2015). Antagomirs bind on the 3’ untranslated region of targeted mRNA strands, which is expected to avoid other miRNAs positioning as to inhibit miRNAs function (Krützfeldt et al., 2005).

ASOs have been shown to be effective in some instances. Antagomirs have been used to target miR-328 in the setting of DMED (Li et al., 2017). Systemic dosing of these antagomirs has been demonstrated to inhibit miR-328 activity in the penis and reduce advanced glycation end products to improve erectile function. However, because diseases are pleiotropic and diverse in their biology, silencing a single miRNA may not always be adequate. Recent studies in this field reveal that single ASOs targeting multiple miRNAs can suppress several miRNAs at once (Lu et al., 2009). A multiple-target anti-miRNA antisense oligodeoxyribonucleotide (MTg-AMO) is the result of this strategy. In a wide variety of malignancies, miR-21, miR-155 and miR-17-5p are overexpressed, and one MTg-AMO was created to target these three oncogenic microRNAs. Individual AMOs that target a single miRNA and combinations of AMOs that target several miRNAs were shown to be less effective than this MTg-AMO (Lu et al., 2009). In the future, MTg-AMOs might be engineered to concurrently block the activity of miRNAs that have a role in DMED.

Another novel approach is to develop competitive inhibitors of miRNA activity. Vectors having several artificial miRNA binding sites are called “miRNA sponges”. They are used to create enormous amounts of transcript under the direction of powerful promoters and keep homologous miRNAs from binding to their native targets as a sponge (Cohen, 2009; Hansen et al., 2013). This method was utilized to decrease miR-141 and miR-205 expression, revealing their function in DMED (Wen et al., 2018; Wen et al., 2019).

### Therapies That Restore miRNAs Function

Numerous techniques for reactivating miRNAs with disease-suppressive effects that are downregulated in DMED have been proposed. A new study shows that “miRNA replacement therapy”, a method for restoring miR-874-3p expression in DMED, has been successfully used (Huo et al., 2020a). This miRNA was delivered by an f in a rat model of DMED, which resulted in improved erectile function and decreased apoptosis. Conventional gene therapy procedures, on the other hand, have the same issues in delivering protein-coding genes via viral delivery (Kay, 2011).

A substantial body of research indicates that the majority of human diseases are characterized by miRNA production abnormalities that result in a global decrease in miRNA levels. As a result, a therapeutic benefit might be gained by returning the global miRNAome to a normal state. A new “miRNAome-based” approach has been proposed as a result of these discoveries. By binding to TARBP2, the small chemical enoxacin facilitates RNAi and miRNA processing (Shan et al., 2008). Following therapy with enoxacin, a global restoration of downregulated miRNAs to more normal miRNA expression patterns has been demonstrated to prevent tumor progression (Melo et al., 2011). The medication had no effect on healthy cells and was not hazardous to mice. Other methods for reviving the global miRNAome include the use of histone acetylase inhibitors and DNA demethylating agents. Epigenetic silencing of diseases-suppressive miRNAs is released by these chemicals, which have demonstrated therapeutic effectiveness and have been approved for the treatment of some hematological malignancies despite their lack of target specificity (Rodríguez-Paredes and Esteller, 2011; Zhang and Zhang, 2020). However, no comparable research exists in DMED up to now.

### Targeting Other Types of ncRNAs

It’s possible that similar techniques outlined before for improving deregulated miRNAs may be applicable to additional ncRNAs, hence expanding the therapeutic target pool. Even while lncRNAs can be targeted using siRNAs (Zhang et al., 2021), they are more challenging to block than miRNAs because of their complex secondary structures (Tsai et al., 2010). Our growing understanding of other ncRNAs is also being leveraged to generate innovative therapeutic strategies for a variety of diseases (Matsui and Corey, 2017). While further research is necessary, cell lines, mouse models and non-human primate investigations have yielded encouraging findings so far. Clinical applications of ncRNAs-based therapeutics are still a long way off, but researchers are optimistic.

## Perspectives for ncRNAs in Diabetes Mellitus Induced Erectile Dysfunction

There has been considerable interest in ncRNAs recently, but more research is needed to properly understand their function and the mechanisms through which they exert their effects. One significant hurdle will be identifying all of the human genome’s functional ncRNAs, for which developing genomic, epigenomic, and bioinformatic techniques will be critical. ENCODE, for example, is making significant progress in its mission to catalog all of the human genome’s functional components (Birney et al., 2007; Encode Project Consortium, 2012). Second-generation sequencing methods, like RNA sequencing, will yield a more complete view of the human ncRNA transcriptome (Pareek et al., 2011). The use of bioinformatics methods to find ncRNAs that may be useful will also be critical (Chen et al., 2019). Due of the complex secondary structures that ncRNAs form to function, sequence-based alignments alone may be insufficient to detect ncRNAs. Albeit plenty of algorithms have been established to predict ncRNAs with potential function (Wang et al., 2020; Singh et al., 2021; Fu et al., 2022), only a few kinds of ncRNAs can be identified so far.

The identification of ncRNA defects in human diseases has boosted expectations in the therapeutic field. Inhibition of crucial DMED-promoting genes that were previously not regarded feasible conventional therapeutic targets is now possible with siRNAs or miRNAs, which expand the universe of “druggable” targets. Clinical studies utilizing compounds derived from ncRNAs are currently being conducted. However, novel techniques, such as small molecule inhibitors of the miRNA machinery, are on the horizon. Many hurdles must be overcome before this strategy may be beneficial in treating DMED in a way that standard treatments haven't yet been able to. Furthermore, except for miRNAs and lncRNAs, there are currently no research examining the involvement of additional ncRNAs in DMED. New discoveries are predicted to be made as the field evolves. Exciting times lie ahead for us (Esteller, 2011).

## Conclusion

Since their identification as functional RNAs, ncRNAs have been regarded as the milestone in the treatment of numerous diseases, even though therapeutic applications are still in their infancy. Similarly, the discovery of ncRNAs has ushered in a new age in DMED, one that may provide a slew of novel biomarkers or therapeutic targets and revolutionize how DMED is diagnosed and treated.
